# Investigation of the anatomic risk factors in acute anterior cruciate ligament ruptures to develop ramp lesions of the medial meniscus by quantitative MRI

**DOI:** 10.1186/s13244-024-01685-w

**Published:** 2024-06-03

**Authors:** Ziyi Tang, Yuxi Luo, Dan Liu, Suying Zhou, Zhangyan Xu, Tongxin Zhu, HaiTao Yang

**Affiliations:** Department of Radiology, the First Affiliated Hospital of Chongqing Medical University, Chongqing, 400016 China

**Keywords:** ACL ruptures, Ramp lesions, Knee, Regional anatomy, Magnetic resonance imaging

## Abstract

**Objective:**

To investigate the anatomic risk factors of knee in patients with acute non-contact anterior cruciate ligament (aACL) ruptures to develop ramp lesions.

**Methods:**

A total of 202 subjects were retrospectively divided into three groups: (1) aACL ruptures combined with ramp lesions group (*n* = 76); (2) isolated ACL ruptures group (*n* = 56) and (3) normal controls group (*n* = 70). Quantitative morphological parameters on MRI were measured including: diameter of medial femoral condyle (MFC), anterior-posterior length and depth of medial tibial plateau (MTP AP length and depth), lateral posterior tibial slope (LPTS) and medial posterior tibial slope (MTPS), asymmetry of LPTS and MPTS (LMPTS), lateral meniscal slope (LMS), and medial meniscal slope (MMS).

**Results:**

The MTP AP length, MTP AP length/MFC diameter ratio, MTP depth, LPTS and the asymmetry of LMPTS showed significant differences among the three groups (*p* < 0.001). The risk factors associated with the ramp lesions including a longer MTP AP length (OR 1.17, 95% CI 1.00–1.44, *p* = 0.044), increased MTP depth (OR 1.91, 95% CI 1.22–3.00, *p* = 0.005) and lager ratio (OR 1.11, 95% CI 1.01–1.22, *p* = 0.036). The highest AUC was the MTP AP length/MFC diameter ratio (0.74; 95% CI, 0.66–0.82). The combination model increased higher accuracy (0.80; 95% CI, 0.72–0.88).

**Conclusion:**

Several bony anatomic characteristics of the knee, especially the morphology of medial tibia plateau, are additional risk factors for aACL ruptures to develop ramp lesions.

**Critical relevance statement:**

Predictive anatomic risk factors of the knee for patients with acute non-contact anterior cruciate ligament (aACL) ruptures to develop ramp lesions, especially the morphology of medial tibia plateau, are detectable by MRI.

**Key Points:**

- Ramp lesion development can complicate aACL ruptures and requires specific treatment.
- Longer AP length and increased MTP depth are risk factors for concurrent ramp lesions.
- Identification of ramp lesions allows for the most appropriate treatment.

**Graphical Abstract:**

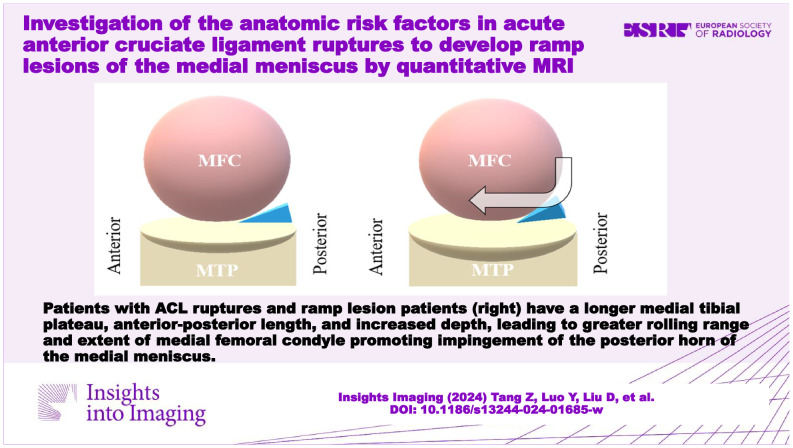

## Introduction

Among knee injuries, anterior cruciate ligament (ACL) injuries occur in about 50% of patients, of which about 3/4 are non-contact injuries. These injuries can could cause knee instability and accelerate the progression of arthritis [1, 2]. The occurrence of meniscal tears is a common combined injury found in up to 61% ofacute ACL injuries. [3] The posterior horn of the medial meniscus (PHMM) serves as a secondary restraint against both anterior and posteromedial rotation of the tibia [3–6]. Known as peripheral lesions less than 2.0 cm, ramp lesions represent a particular form of meniscus injury involving the meniscocapsular or meniscosynovial attachments and red-red zone of the PHMM [7–10]. The incidence of ramp lesions in patients with ACL injury ranges from 9–34.5% [11–13]. However, due to their concealed location, the rate of missed diagnosis is alarmingly high (40% with arthroscopy and over 50% with magnetic resonance imaging (MRI)) [14–17]. Previous studies have underscored that only reconstructing the ACL without addressing concurrent ramp lesions may result in persistent joint instability, and potentially hasten the degeneration of the meniscus and cartilage, eventually leading to failure of the reconstructed ACL and poorer prognosis [4, 8, 13, 15, 18]. Therefore, it is critical to accurately identify the combined ramp lesions in patients with ACL injury including its anatomical, biomechanical and diagnostic features.

Bone contusion at the posterior medial tibial plateau, along with chronic ACL injury, steeper medial tibial and meniscal slope, gradual lateral tibial slope, and varus knee > 3° have been reported as potential risk factors for ramp lesions [12, 19–21]. However, ramp lesions can occur during traumatic acute ACL injury or increased tibial translation in chronic ACL-deficient knee. Previous studies have shown that the incidence of ramp lesions markedly increases with the extended ACL injury time, and might be considered a secondary injury after ACL rupture [8, 12, 22]. The acute non-contact anterior cruciate ligament (aACL) ruptures coexisted with ramp lesions of the medial meniscus is a severe combined injury and may have different anatomical risk factors or biomechanical patterns. However, there is a scarcity of studies examining the anatomical risk factors or biomechanical mechanisms contributing to aACL ruptures leading to ramp lesions. Previous research has delved into the anatomical morphology of the medial knee compartment in isolated ACL ruptures and pure ramp lesions [12, 23, 24]. Therefore, we hypothesized that certain bony variations within the medial tibiofemoral joint might be linked to the development of ramp lesions in conjunction with aACL ruptures. Understanding these mechanisms could provide insights for treatment and prevention strategies for acute combined injuries. Thus, the primary objective of this study was to identify predictive anatomical risk factors associated with aACL ruptures coexisting with ramp lesions of the medial meniscus.

## Methods

### Subjects

Before the research was started, this retrospective comparative study received institutional review board approval. We retrospectively reviewed the clinical record and MRI images of the patients diagnosed with ACL rupture from January 2013 to May 2023 by using the Picture Archiving and Communication System (PACS) and Hospital Information System (HIS) at our hospital by one musculoskeletal radiologist (Tang, with 3 years of clinical experience). Patients were assigned to the following 3 groups: (1) aACL ruptures combined with ramp lesions, (2) isolated aACL ruptures, and (3) a normal control group.

**Fig. 1:**
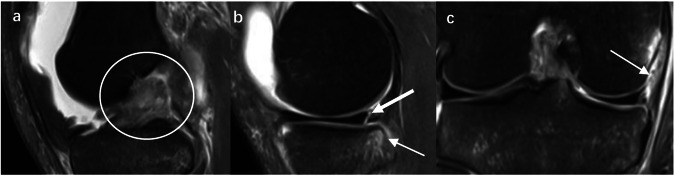
Diagnosis of aACL rupture combined with ramp lesions on MRI. A 27-year-old male sprained right knee while playing basketball with knee pain for 5 days. Sagittal and coronal IM-WI MR images with fat saturation showed acute ACL complete rupture (**a**, circle); the complete fluid filling signal at the meniscocapsular junction of PHMM (**b**, bold arrow); the bone contusion on the posterior of MTP (**b**, pinpointed arrow) and swelling of the meniscofemoral ligament (**c**, arrow). PHMM, posterior horn of the medial meniscus; MTP, medial tibial plateau; IMWI, intermediate weighted image

The inclusion criteria for Group 1 were as follows: (1) confirmation of aACL rupture combined with ramp lesions through arthroscopic surgery; (2) and/or presence of the typical characteristics on MRI defined as the complete fluid filling signal at the meniscocapsular junction of PHMM at least two slices on sagittal images (Fig. 1) [25, 26]; (3) patients with a well-defined history of non-contact knee injury occurring within the past 3 months. The early included patients were not confirmed with ramp lesions under arthroscopy, replacing those confirmed by the typical MRI manifestations mentioned above, due to orthopedic surgeons being insufficiently aware of ramp lesions and missing the diagnosis in the early period of our data duration. All patients diagnosed by MRI were confirmed by a senior musculoskeletal radiologist (Yang, with 20 years of clinical experience). As for Group 2, the inclusion criteria were: (1) confirmation of isolated aACL ruptures without concurrent meniscal tears or ligament injuries through arthroscopic surgery; (2) patients with a clear history of non-contact knee injury within the past 3 months. Subjects in the normal group were selected from among those who showed normal knee MRI manifestation without any detectable abnormalities or structural lesions.

**Fig. 2:**
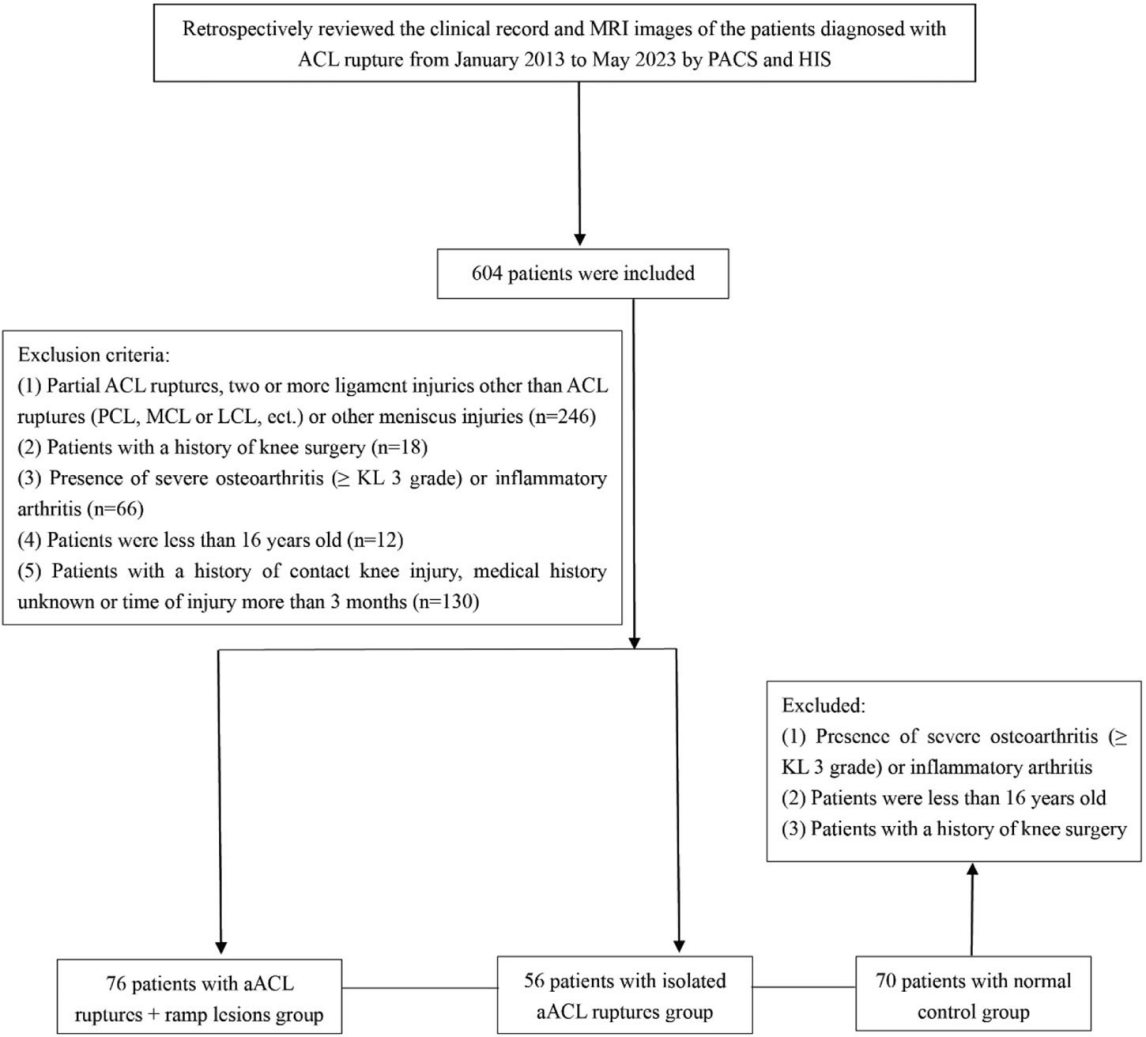
The patient flowchart of the study. aACL, acute non-contact anterior cruciate ligament; PCL, posterior cruciate ligament; MCL, medial collateral ligament; LCL, lateral collateral ligament

Exclusion criteria were as follows: (1) partial ACL ruptures or additional other ligamental tears with ACL ruptures (such as posterior cruciate ligaments (PCL), medial and lateral collateral ligaments (MCL or LCL), etc.); (2) patients with a history of knee surgery; (3) presence of severe osteoarthritis (≥ KL 3 grade) or inflammatory arthritis; (4) patients under the age of 16; and (5) patients with a history of contact knee injury, unknown medical history, or the time from injury to MRI examination more than 3 months. All subjects in the normal control group met the exclusion criteria and accepted MR scanning during the same period and of a similar age range at our department. In cases where the diagnosis on MRI was uncertain, a final determination was made by a senior musculoskeletal radiologist (Yang, with 20 years of clinical experience). Following to review of clinical and imaging data from PACS and HIS, the patients in three groups meeting above criteria were enrolled in this study. A flowchart of the patient recruitment process is shown in Fig. 2.

### MRI protocol

Magnetic resonance imaging was performed using 1.5 or 3.0-T MRI equipment (Magnetom Essenza or Skyra; Siemens Healthcare, Munich, Germany) with an extremity matrix knee coil. The imaging protocol was as follows: sagittal and coronal turbo spin echo (TSE) T1-weighted sequence (repetition time/echo time 550/11-13 ms, field of view 16 cm, matrix size 320 × 320, slice thickness 4 mm), sagittal TSE T2-weighted sequence (repetition time/echo time 3000–3350/44–116 ms, field of view 16 cm, matrix size 320 × 320, slice thickness 4 mm), sagittal and coronal TSE intermediate weighted sequence with fat saturation (repetition time/ echo time 2200–3000/39–48 ms, field of view 16 cm, matrix size 256 × 256, slice thickness 4 mm), and axial TSE intermediate weighted sequence with fat saturation (repetition time/ echo time 2790–3200/26–67 ms, field of view 16 cm, matrix size 256 × 256, slice thickness 4 mm). Most of the scanning parameters were similar between 1.5- and 3.0-T MRI equipment to keep the consistency of image quality. We focused on detecting the macro structures including bone, ligament and meniscus rather than micro structures such as cartilage or synovium. Thus, we considered there was no influence of the MRI field strength in evaluating the main structure of this study, which is similar to previous studies [27, 28].

### Quantitative MRI measurements

**Fig. 3:**
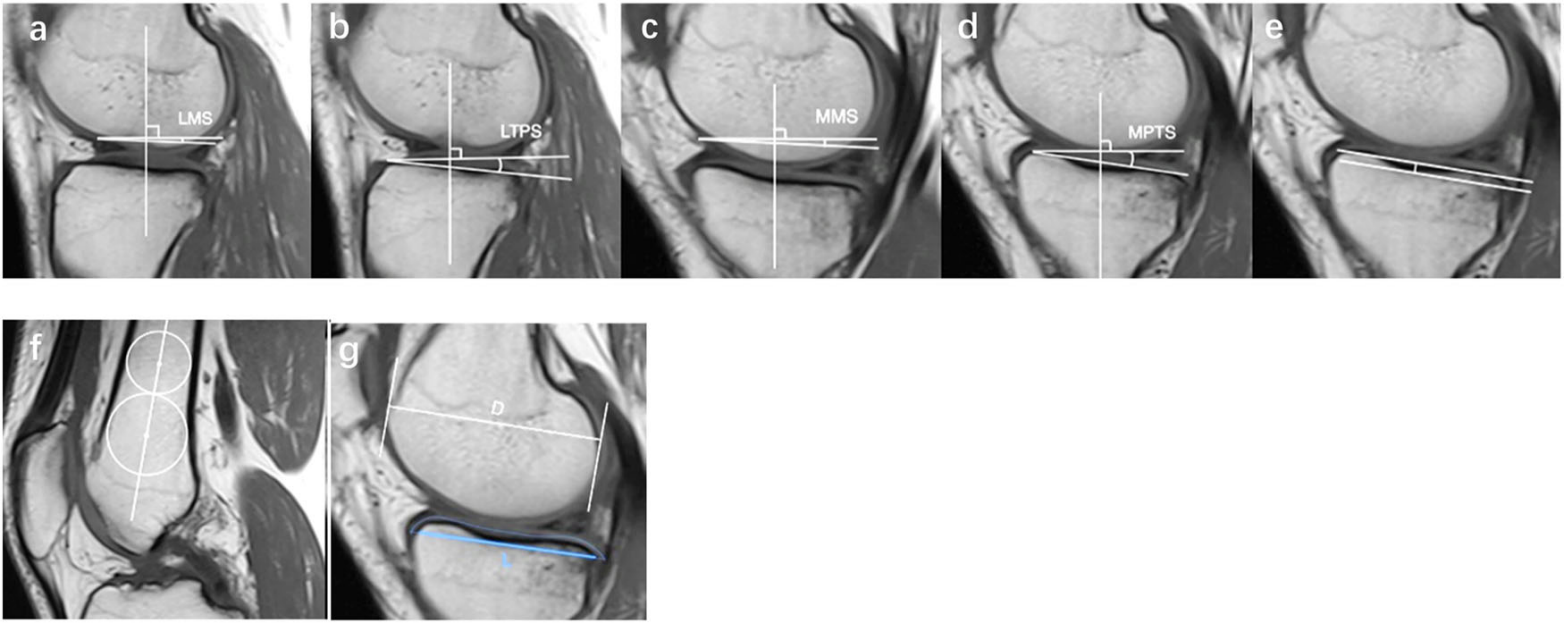
Quantitative MRI measurements of the knee. **a** LMS, lateral meniscal slope; **b** LPTS, lateral posterior tibial slope; **c** MMS, medial meniscal slope; **d** MTPS, medial posterior tibial slope; **e** MTP depth, medial posterior tibial depth; **f** drawing two circles tangential to the cortex in the femoral shaft on the mid-sagittal MRI, then the femoral longitudinal axis was established by connecting the midpoints of these two circles; **g** lines paralleling to the femoral longitudinal axis were drawn tangential to both anterior-most and posterior-most aspects of the MFC; the MFC diameter was determined as the distance between these two lines (D). An outline of the cartilaginous surface of the MTP was delineated, and a line connecting its anterior and posterior edges was considered as the subchondral surface of the MTP, and intersection points were marked where this line intersected with the anterior and posterior edges of the MTP subchondral bone, defining their distance as MTP AP length (L). MFC, medial femoral condyle; MTP AP length, medial tibial plateau anterior-posterior length

Four parameters were applied to quantitative evaluation. The lateral posterior tibial slope (LPTS) and medial posterior tibial slope (MTPS) on MRI were manually measured referring to previous studies by Hudek et al [19] and Song et al [22] (Fig. 3b, d), and the asymmetry of the LPTS and MPTS (LMPTS) was defined as LPTS minus MPTS. The measurement of the MTP anterior-to-posterior length (MTP AP length) was adapted and employed in this study by Yoshihara et al [29] at the central sagittal MRI slice of the MTP (Fig. 3g). In this method, (1) outlining the cartilaginous surface of the MTP and considering a line connecting its anterior and posterior edges as the subchondral surface, and (2) marking the intersection points where this line intersected with the anterior and posterior edges of the MTP subchondral bone, defining their distance as MTP AP length. The MTP depth (Fig. 3e) was measured at the same slice described by Hashemi et al [30] In addition, the lateral meniscal slope (LMS) and medial meniscal slope (MMS) on MRI were also manually measured referring by previous studies to Hudek et al [19] and Song et al [22] (Fig. 3a, c).

For the parameters of femoral condyle, the diameter of the medial femoral condyle (MFC) was assessed at the central sagittal plane, following the method described by Yoshihara et al (Fig. 3f, g) [29]. This involved the following steps: (1) drawing two circles that were tangential to the cortex in the femoral shaft on the mid-sagittal MRI; subsequently, the femoral longitudinal axis was established by connecting the midpoints of these two circles. Next, this axis was projected onto the MRI sagittal slice at the medial-to-lateral midpoint of the MFC; (2) lines paralleling to the femoral longitudinal axis were then drawn tangential to both the anterior-most and posterior-most aspects of the MFC. The MFC diameter was determined as the distance between these two lines. To account for variations in bone size among individuals, we calculated a ratio between the MTP AP length and the MFC diameter.

All MRI parameters were quantitatively measured by two trained musculoskeletal radiologists (Tang and Liu, with 3 and 6 years of clinical experience), and the interval between the first and second reading was 4 weeks (Tang). Before quantitative MRI measurements, two assessors underwent systematic training and practice for understanding the MRI features of the each of lesions and methods, and needed to pass the test by a senior musculoskeletal radiologist (Yang).

### Statistical analysis

Statistical analyses were performed with SPSS software (IBM Corp. Released 2019. IBM SPSS Statistics for Windows, Version 26.0. Armonk, NY: IBM Corp). All the measurement parameters of the knee were first checked for normal distribution by the Kolmogorov‒Smirnov test. The parameters of the normal distribution were used by one-way ANOVA test to compare the difference among the three groups, while the parameters of the non-normal distribution were used by Kruskal-Wallis one-way ANOVA test. A Bonferroni correction with a *p*-value less than 0.05/n (*n* = 3) was applied as statistically significant to control the overall significance level for multiple hypothesis testing. For the parameters with statistical differences, multivariable logistic regression analysis was further used to analyze the independent predictors of the aACL ruptures with ramp lesions group from the isolate aACL ruptures group. Receiver operating characteristic (ROC) curves and the area under the curve (AUC) were constructed to evaluate the diagnostic accuracy of the valuable quantitative parameters with the cutoffs calculated. The ideal predictive cutoff point with the highest sensitivity and specificity was determined by the Youden index. Intra-class correlation coefficients (ICCs) were used to determine the intra- and inter-reader agreement of these measurements and classified as good (≥ 0.75), fair (0.50–0.74) and poor (< 0.50). Significance was accepted with a *p-*value of less than 0.05 except for the statistical tests with a Bonferroni correction.

## Results

### Demographic characteristics of subjects in three groups

Among 604 patients, 76 patients (63 men, 13 women, mean age 27.5 ± 7.3 years) of aACL ruptures combined with ramp lesions, and 56 patients (40 men, 16 women, mean age 29.6 ± 8.2 years) of isolated aACL ruptures were retrieved into the two cases group. An additional 70 patients (58 men, 12 women, mean age 29.7 ± 7.8 years) of normal knee structures were enrolled in the control group. The incidence of ramp lesions in aACL ruptures was 17.4%. The patient characteristics according to the presence of ramp lesions are summarized in Table 1. There were no significant differences in gender, age, and injured side among the three groups.

**Table 1:** Patients and injury characteristics

	aACL ruptures + ramp lesions	aACL ruptures		Control group	*p* value
No. of patients	76	56		70	
Age	27.5 ± 7.3	29.6 ± 8.2		29.7 ± 7.8	0.131
Gender					0.194
Man	63 (82.9)	40 (71.4)		58 (82.9)	
Woman	13 (17.1)	16 (28.6)		12 (17.1)	
Injured side					0.508
Left	42 (55.3)	26 (46.4)		33 (47.1)	
Right	34 (44.7)	30 (53.6)		37 (52.9)	

Values are presented as *n* (%) or mean ± SD

*p* value for age by Kruskal-Wallis one-way ANOVA test*; p* value for gender, affected side by Chi-square test

*aACL* acute non-contact anterior cruciate ligament

### Comparisons of the quantitative MRI measurements among groups

The MTP AP length, MTP AP length/MFC diameter ratio, MTP depth, LPTS and the asymmetry of LMPTS showed significant differences among the three groups (*p* < 0.001), while no differences were in the MFC diameter, MPTS, LMS and MMS. The MTP AP length was significantly longer (40.9 ± 3.7), the MTP AP length/MFC diameter ratio was significantly larger (0.87 ± 0.06), the MTP depth was significantly deeper (2.4 ± 0.9), the LPTS (6.42 ± 2.17) was significantly steeper, with an increased the asymmetry of LMPTS (3.38 ± 2.07) in the group of aACL ruptures with ramp lesions. For multiple comparisons among the three groups, the LPTS and the asymmetry of LMPTS showed significant differences between each group. The MTP AP length, MTP AP length/MFC diameter ratio and MTP depth displayed significant differences in the sub-comparison between the aACL ruptures combined with ramp lesions group and the isolated aACL ruptures group, and between the aACL ruptures combined with ramp lesions group and the control group, while no difference between the isolated aACL ruptures group and the control group (Table 2).

**Table 2:** Quantitative MRI measurements among groups

	aACL ruptures + ramp lesions	aACL ruptures	Control group	*p* value	*p*^a^ value	*p*^b^ value	*p*^c^ value
MFC diameter (mm)	47.2 ± 3.1	46.2 ± 3.3	46.2 ± 3.0	0.071	—	—	—
MTP AP length (mm)	40.9 ± 3.7	37.8 ± 3.1	37.7 ± 2.5	< 0.001	< 0.001	< 0.001	0.921
MTP AP length/MFC diameter ratio	0.87 ± 0.06	0.82 ± 0.06	0.83 ± 0.05	< 0.001	< 0.001	< 0.001	> 0.999
MTP depth (mm)	2.4 ± 0.9	1.8 ± 0.9	1.7 ± 0.7	< 0.001	0.001	< 0.001	> 0.999
LPTS (°)	6.42 ± 2.17	5.44 ± 2.57	4.02 ± 1.74	< 0.001	0.032	< 0.001	0.001
MPTS (°)	3.03 ± 1.54	3.00 ± 1.79	2.55 ± 1.29	0.103	—	—	—
The asymmetry of LMPTS (°)	3.38 ± 2.07	2.43 ± 2.34	1.48 ± 1.89	< 0.001	0.032	< 0.001	0.047
LMS (°)	4.50 ± 2.40	3.92 ± 2.35	3.39 ± 1.42	0.052	—	—	—
MMS (°)	3.20 ± 1.76	3.13 ± 1.63	2.77 ± 1.32	0.382	—	—	—

*p* value from the one-way ANOVA test or Kruskal-Wallis one-way ANOVA test. For multiple comparisons among the three groups, a Bonferroni correction was applied. Values are presented as mean ± SD

Bolded values indicate statistical significance

*p* value was defined between the ACL ruptures + ramp lesions group, the ACL ruptures group and the control group

*p*^a^ value was defined between the ACL ruptures + ramp group and the ACL ruptures group

*p*^b^ value was defined between the ACL ruptures + ramp group and the control group

*p*^c^ value was defined between the ACL ruptures group and the control group

*aACL* acute non-contact anterior cruciate ligament, *MFC* medial femoral condyle, *MTP AP* length medial tibial plateau anterior-posterior length, *LPTS* lateral posterior tibial slope, *MTPS* medial posterior tibial slope, *the asymmetry of LMPTS* the asymmetry of LPTS and MPTS, *LMS* lateral meniscal slope, *MMS* medial meniscal slope

### Correlation of quantitative MRI measurements with ramp lesions

The factors associated aACL ruptures with concurrent ramp lesions were identified and are summarized in Table 3. Risk analysis showed that a longer MTP AP length (OR 1.17, 95% CI 1.00–1.44, *p* = 0.044), increased MTP depth (OR 1.91, 95% CI 1.22–3.00, *p* = 0.005) and lager MTP AP length/MFC diameter ratio (OR 1.11, 95% CI 1.01–1.22, *p* = 0.036) increased the presence of ramp lesions in aACL ruptures knees.

**Table 3:** Multivariable logistic regression analysis: association between the presence of ramp lesions and morphological risk factors

	Odds Ratio	95% CI	*p* value
MTP AP length (mm)	1.17	1.00–1.44	**0.044**
MTP depth (mm)	1.91	1.22–3.00	**0.005**
MTP AP length/MFC diameter ratio	1.11	1.01–1.22	**0.036**
LPTS (°)	1.15	0.89–1.48	0.294
The asymmetry of LMPTS (°)	1.07	0.81–1.40	0.649

Bolded values indicate statistical significance

*MFC* medial femoral condyle, *MTP AP length* medial tibial plateau anterior-posterior length, *LPTS* lateral posterior tibial slope, *the asymmetry of LMPTS* the asymmetry of LPTS and MPTS

### Comparisons of the diagnostic efficiency of the quantitative MRI measurements

**Fig. 4:**
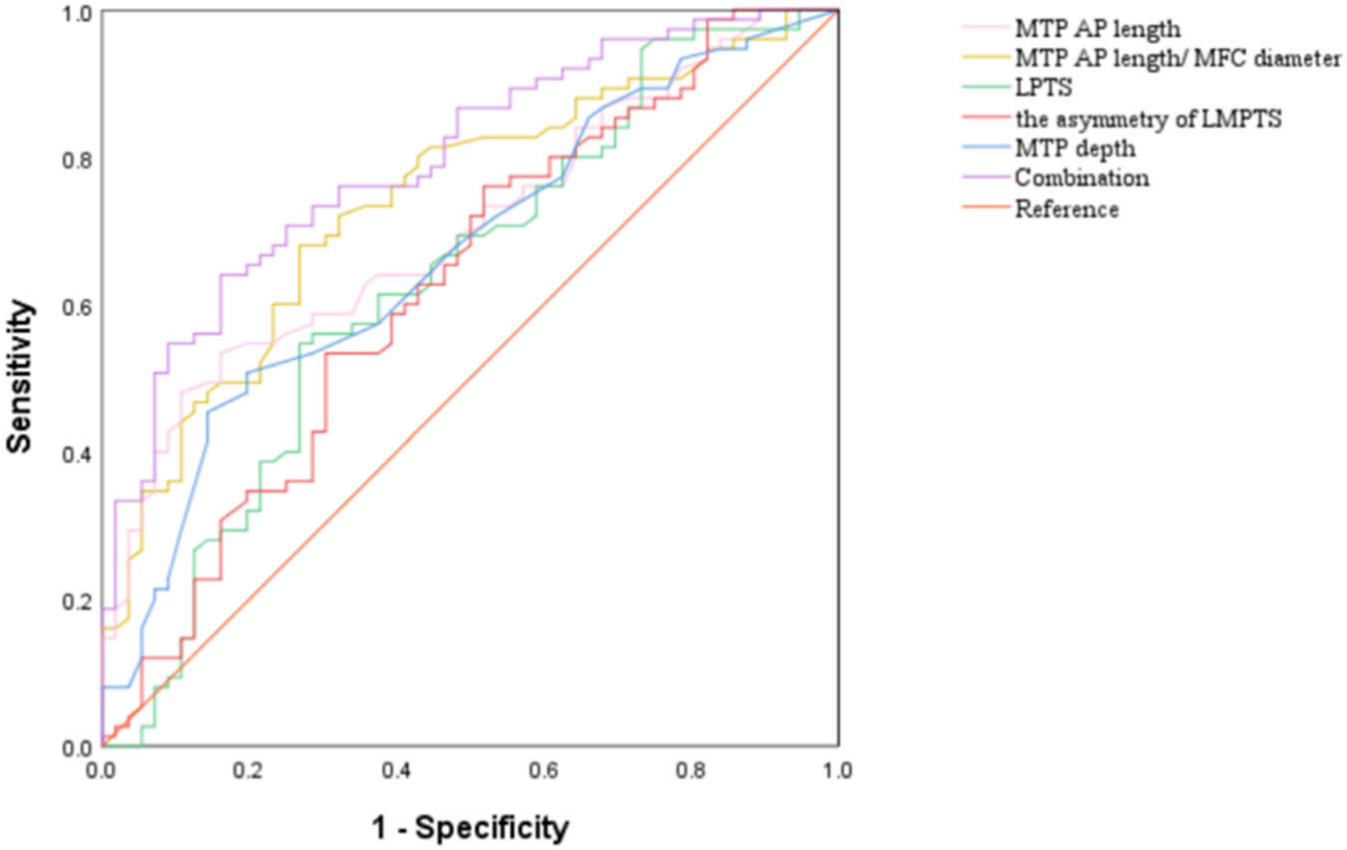
Receiver operating characteristic curves of each morphological risk factor for ramp lesions

ROC curve and AUC analyses of the associated quantitative measurements are shown in Table 4 and Fig. 4, respectively. The MTP AP length, MTP depth, MTP AP length/MFC diameter ratio, LPTS and the asymmetry of LMPTS showed significant differences in the comparison of diagnostic efficiency. The highest AUC was MTP AP length/MFC diameter ratio (0.74; 95% CI, 0.66–0.82), with a sensitivity of 73% and specificity of 68% to predict the ramp lesions and the calculated cutoff value was 0.84. The combination of the MTP AP length, MTP depth, MTP AP length/MFC diameter ratio, LPTS and the asymmetry of LMPTS via logistic regression increased higher accuracy (0.80; 95% CI, 0.72–0.88), with a sensitivity of 84% and specificity of 64%.

**Table 4:** Diagnostic performance among all MRI measurements

	MTP AP length	MTP depth	MTP AP length/ MFC diameter ratio	LPTS	the asymmetry of LMPTS	Combination
Sensitivity	0.89	0.85	0.73	0.73	0.48	0.84
Specificity	0.48	0.45	0.68	0.54	0.75	0.64
Cut off value	41.1	2.55	0.84	6.46	1.91	0.61
AUC	0.70	0.67	0.74	0.63	0.63	0.80
AUC:95%	0.62–0.79	0.58–0.76	0.66–0.82	0.53–0.73	0.53–0.73	0.72–0.88
*p* value	< 0.001	0.001	< 0.001	0.010	0.013	< 0.001

*p* value for AUC

*AUC* area under curve, *MFC* medial femoral condyle, *MTP AP length* medial tibial plateau anterior-posterior length, *LPTS* lateral posterior tibial slope, *the asymmetry of LMPTS* the asymmetry of LPTS and MPTS

### Intra- and inter-reader reliability of quantitative MRI measurements

For the associated measurements, the intra-reader ICCs for the quantitative measurements ranged from 0.775 to 0.957, whereas the inter-reader ICCs ranged from 0.765 to 0.944, indicating that raters were in good agreement.

## Discussion

This study investigated the anatomic risk factors of aACL ruptures to develop ramp lesions by comparing the anatomic morphological characteristics including the meniscal and tibial slope, MFC and the MTP AP length and depth among the aACL ruptures combined with ramp lesions, isolated aACL ruptures and normal groups by quantitative MRI. The most important finding of the study was that patients with aACL ruptures combined with ramp lesions presented a longer MTP AP length, increased MTP depth, and lager MTP AP length/ MFC diameter ratio compared with isolated aACL ruptures and normal knees.

Anatomic variations of bone and soft tissue in the lateral and medial compartments of the knee have been identified as risk factors for isolated ACL ruptures or ramp lesions [12, 19, 22, 23]. Nonetheless, very limited knowledge about the role of these variations in the aACL ruptures developing concurrent ramp lesions. First, we investigated several anatomic factors reported in previous studies including the lateral and medial posterior tibial slope (LPTS and MPTS), the asymmetry of LMPTS, medial and lateral meniscal slope (MMS and, LMS) and medial femoral condyle (MFC). LPTS has been extensively inspected as a potential risk factor for non-contact ACL injuries [19, 23, 24, 29], but few studies have assessed whether it is related to ramp lesions. A cross-sectional study has indicated that the LPTS and the asymmetry of LMPTS were risk factors for ramp lesions in ACL-injured knees [12], while another study reported no correlation between LPTS and ramp lesions [22]. Our study showed that there were differences in both the LPTS and the asymmetry of LMPTS between each of the groups. Biomechanical experiments have indicated that the pivot-shift mechanism plays a pivotal role in ACL rupture, with steeper LPTS and higher asymmetry of LMPTS contributing to increased anterior translation and internal rotation of the tibia [23, 31–34]. Our findings demonstrated a progressively increased trend of LPTS and the asymmetry of LMPTS across the three groups, and the most pronounced features were displayed in the aACL ruptures combined with ramp lesions group with the steepest LPTS and greatest asymmetry of LMPTS. It suggests that a steeper LPTS and increased asymmetry of LMPTS may be associated with a higher likelihood of ramp lesions. Consistent with previous studies, there were no differences among the three groups for the MPTS, MFC diameter and LMS [12, 22, 23]. MMS also showed no difference among the three groups, which was somewhat inconsistent with the previous studies. Several studies have indicated that increased MMS was an independent anatomical risk factor for ramp lesions, particularly in patients over 6 months since the time of injury [12, 22]. This inconsistency may be attributed to our research objects focusing on patients with acute ACL injury. When the meniscal slope becomes steeper, the slide range of the femoral condyle increases and leads to ACL gaining greater stress and rupture [12, 22]. However, when combined with ramp lesions, the PHMM loses its stability and may shift anteriorly, resulting in changes to the meniscal slope. With the extension of injury durations, the PHMM slides forward and deeply into the medial tibia plateau, and then the MMS becomes steeper than its original place. Therefore, we assume that the increase of MMS is more likely to be a result of ramp lesions than a risk factor. Certain bony factors in the medial compartment of the knee need to further explore the potential risks.

Our study revealed two risk factors of the MTP for ramp lesions in aACL ruptures including a longer MTP AP length and increased MTP depth, and was the first to observe that the MTP AP length was significantly longer, the MTP AP length/ MFC diameter ratio was larger, and the MTP depth was deeper in the aACL ruptures with ramp lesions. Previous studies mainly focused on MMS and MPTS of the medial compartment for assessing the risk factors of ramp lesions [12, 22]. Some studies have found that a biconcave MTP is more frequently associated with complex medial meniscus tears [23, 24, 31, 33]. Increased MTP depth has been suggested to enhance joint congruity by deepening the articular surface of the tibial plateau, potentially offering improved resistance against anterior tibial translation [30]. Conversely, a shallow MTP depth may result in impingement of the PHMM and reduced lateral AP translation in healthy knees [31, 33]. It is plausible that a longer MTP AP length, coupled with a deep MTP depth, contributes to an increased scrolling range of the MFC on the MTP, thereby resulting in greater anterior translation of the tibia, and then impinging the PHMM, eventually leading to ramp lesions.

**Fig. 5:**
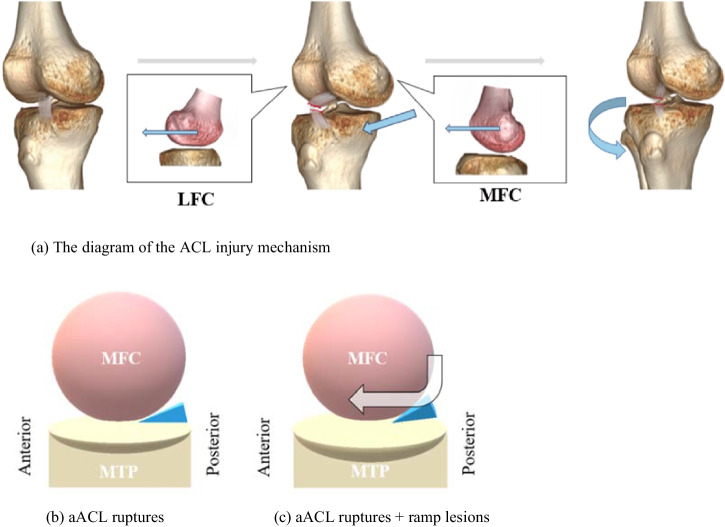
**a** When the ACL ruptures, it leads to anterior translation and internal rotation of the tibia, meanwhile, posterior rolling of the LFC and anterior rolling of the MFC. Compared with the isolate aACL ruptures patients (**b**), the aACL ruptures + ramp lesion patients (**c**) have a longer MTP AP length and increased MTP depth, which would lead to the greater rolling range and extent of MFC and then easily impinge the PHMM. aACL, acute non-contact anterior cruciate ligament; MFC, medial femoral condyle; MTP AP length, medial tibial plateau anterior-posterior length; PHMM, posterior horn of the medial meniscus; LFC, lateral femoral condyle

When the ACL ruptures, the compressive forces result in an anteriorly directed shear force, eventually leading to anterior translation and internal rotation of the tibia, posterior rolling of the LFC, and anterior rolling of the MFC, relatively [2, 29, 35, 36]. According to previous studies, a steeper LPTS and a higher asymmetry of LMPTS could further increase anterior translation and internal rotation of the tibia. The findings of our study suggest that a longer MTP AP length and increased MTP depth would lead to a greater rolling range and extent of MFC and then easily impinge the PHMM. Additionally, possibly adding the contraction of the semimembranosus tendon to pull back the PHMM with a posteriorly directed shear force [37, 38], consequently developing ramp lesions in aACL ruptures (Fig. 5).

Our study has some limitations. First, due to the wide range of years during which patients were collected, not all cases included in our study were diagnosed with ramp lesions under arthroscopy as orthopedic surgeons might be insufficiently aware of ramp lesions,missing diagnoses in the early period of our data duration. However, the ramp lesions exhibited the typical MRI characteristics with the complete fluid filling signal at the meniscocapsular junction of PHMM, with specificity as high as 98% in previous studies [14, 25]. Second, some individual factors such as gender, age, and BMI were not considered in our study, but the statistical results showed no difference in gender and age among the three groups. Our research focused on aACL ruptures patients and also excluded the patients with severe osteoarthritis and inflammatory arthritis, which may limit the generalizability of the findings to a broader population. Third, both of these MRI parameters were measured manually. We evaluated them at the central sagittal plane, which was subjective and might not always align precisely with the central sagittal plane. To maintain consistency in slice selection to the best extent possible, we adopted the method proposed by Holdel et al [31] where the midsagittal plane is defined at the level where the popliteal groove is most prominent on the coronal slide. Future work should include multi-center patients and conduct a prospective study combined with clinical function assessments to acknowledge the study’s results and develop a comprehensive understanding of the relationship between aACL ruptures and ramp lesions.

## Conclusion

In general, ramp lesions associated with aACL rupture result from a series of complex mechanisms and are commonly overlooked in preoperative clinical practice. Our study confirmed that some occult anatomic characteristics, especially the morphology of medial tibia plateau, can be additional risk factors for this acute combined injury and produce a potential impact on injury patterns. This may be helpful to further explore the biomechanical mechanism of the aACL ruptures to develop ramp lesions, and trigger some attention to the medial tibial plateau in the treatment and prevention of these lesions.

## Data Availability

All data generated or analyzed during this study are included in the Picture Archiving and Communication System (PACS) and Hospital Information System (HIS) at the First Affiliated Hospital of Chongqing Medical University.

## Abbreviations

aACL: Acute non-contact anterior cruciate ligament
AUC: Area under curve
LMS: Lateral meniscal slope
LPTS: Lateral posterior tibial slope
MFC: Medial femoral condyle
MMS: Medial meniscal slope
MTPS: Medial posterior tibial slope
MTP AP length: Medial tibial plateau anterior-posterior length
the asymmetry of LMPTS: The asymmetry of LPTS and MPTS
PHMM: The posterior horn of the medial meniscus
TSE: Turbo spin echo
